# Mucoadhesive Particles: A Novel, Prolonged-Release Nanocarrier of Sitagliptin for the Treatment of Diabetics

**DOI:** 10.1155/2019/3950942

**Published:** 2019-11-03

**Authors:** Nagaraja SreeHarsha, Chandramouli Ramnarayanan, Bandar E. Al-Dhubiab, Anroop B. Nair, Jagadeesh G. Hiremath, Katharigatta N. Venugopala, Roopashree T. Satish, Mahesh Attimarad, Arshia Shariff

**Affiliations:** ^1^Department of Pharmaceutical Sciences, College of Clinical Pharmacy, King Faisal University, Al-Ahsa, Saudi Arabia; ^2^Department of Pharmaceutics, Vidya Siri College of Pharmacy, Bengaluru, India; ^3^Department of Quality Assurance, Krupanidhi College of Pharmacy, Bengaluru, India; ^4^Department of Biotechnology and Food Technology, Durban University of Technology, Durban 4001, South Africa; ^5^Department of Pharmacognosy, Government College of Pharmacy, Bengaluru, India; ^6^Department of Pharmaceutics, Alard College of Pharmacy, Savitribai Phule, Pune University, Pune, India

## Abstract

Sitagliptin (MK–0431) is a widely and commonly used oral hypoglycemic drug in the treatment of type 2 diabetes mellitus; patients typically take higher doses of this drug (50 mg, twice daily). One drawback is that only 38% of the drug is bound reversibly to plasma proteins and 79% is excreted in urine without being metabolized. To overcome this issue, there is a need for a better drug-delivery method to improve its efficacy in patients. It has been found that in existing formulations, the drug content is 72.5% ± 5% and the percentage yield is 84.9% ± 3%. In this study, sitagliptin nanoparticles (sizes ranging from 210 to 618 nm) were developed. The bioadhesion properties of the nanoparticles, as well as the swelling of the nanoparticles on the mucus membrane aided in sustained drug release. The pattern of drug release was in accordance with the Peppas model. Fourier-transform infrared (FTIR) spectroscopy demonstrated that there were no significant interactions between sitagliptin and chitosan. Differential scanning calorimetry (DSC) results showed an absence of drug peaks due to the fact that the drug was present in an amorphous state. Mucoadhesive nanoparticles were formulated using sitagliptin and were effective for about 12 hours in the gastrointestinal tract. When compared to conventional sitagliptin administration, use of a nanoparticle delivery system demonstrated greater benefits for use in oral delivery applications. This is the first time that a drug-delivery method based on the mucoadhesive properties of nanoparticles could prolong the drug-release time of sitagliptin.

## 1. Introduction

Diabetes represents a complex set of health disorders that affect many patients irrespective of age. Type 2 diabetes mellitus (T2DM) is a form of diabetes that is characterized by the body's inability to produce insulin [1]. Given the widespread nature of this commonly occurring disease, researchers around the world have investigated and developed novel drug-delivery systems that aim to treat it.

The Kingdom of Saudi Arabia is a developing country populated by over 28 million people [2], of which 25% have been diagnosed with diabetes mellitus (DM) [3]. The prevalence of DM has been expected to increase to 40%–50% by 2020 [4]. Medications to treat DM are usually administered orally, and the side effects associated with existing treatments are increased body weight, hypoglycemia, and gastric intolerance. For this reason, T2DM patients remain insufficiently treated. Hence, there has been an immediate need to identify newer therapeutic options for T2DM [5].

Existing medications that were developed to manage DM are characterized by shorter half-lives, meaning that patients must take higher dosages per day, which is not good. Hence, drugs that have prolonged gastric residence times must be formulated to ensure sustained release, which can aid in the prolonged effects of the drug. For this reason, the drug should remain in the upper part of the gastrointestinal tract or in the stomach for longer periods of time. This can only be achieved by sustaining the drug's release until all of the drug is completely released over a desired period of time [6–8]. Existing drugs with prolonged gastric residence times can reveal greater insights into new therapeutic options. Drugs that float and deliver is a concept that was studied by Davis for the first time in 1968, whereby drugs that are bulky in density, and which are lower in density than gastric juice (1.004 g/cm^3^), can remain in the gastrointestinal tract for longer periods, thus being bioavailable and lowering the elimination/degradation of the drug with localized action [9].

When examining the drugs that are available for treating T2DM, sitagliptin (SCN) remains among the best and most effective therapeutic options. In October 2006, the United States Food and Drug Administration (FDA) approved SCN as a basic inhibitor of DPP-4 in the treatment of T2DM. SCN thus falls under the class of DPP-4 inhibitors. It controls HbA1c and fasting glucose levels in patients with T2DM [10]. Many reports have indicated that SCN is a mucoadhesive microsphere with higher gastric retention times [11]. It has been formulated as bilayered tablets with simvastatin [12], trilayered tablets with metformin [13], and gastroretentive floating tablets [14].

DPP-4 inhibitors are keen on extending the actions of endogenous glucagon-like peptide (GLP)-1. The DPP-4 enzyme inhibits proteolysis, thus elevating GLP-1 levels by two to three times, consequently inhibiting >90% of plasma DPP-4 activity in vivo over the course of 24 hours.

Sitagliptin (MK-0431) is involved in inhibiting or delaying incretin degradation. The main functions of DPP-4 inhibitors include mimicking incretin while stimulating insulin secretion, inhibiting glucagon secretion and apoptosis, and ultimately preserving *β*-cell mass. Patients must strictly adhere to dosage intervals of 50 mg twice per day [15–17]. Recently, we have demonstrated the potential of albumin nanoparticles in enhancing a drug's mucoadhesive properties, thereby improving the efficiency of sitagliptin.

Polymeric nanoparticles can effectively alter the pharmacokinetics of drug molecules; they are currently considered the ideal formulations when developing treatments targeting chronic diseases, including diabetes [18]. Despite the fact that hydrogels exhibit highly mucoadhesive properties, they are neutral and weakly interact with the mucosal layer of the membrane [2,4]. However, cationic chitosans, which are copolymers of N-glucosamine, exhibit unique physicochemical and biological properties. In contrast, chitosans, which are cationic, natural copolymers of N-glucosamine, are produced by the deacetylation of chitin and have unique physiochemical and biological properties. Being highly biocompatible, biodegradable, and nontoxic, chitosans are unique to the development of mucoadhesive drug-delivery system (MDDS) and feature special properties [5,6,19,20]. Chitosan has been widely formulated into drug carriers, including nano- to microsized particles [20–22].

An ionic bond between the amino group of chitosans and the sialic acid of mucins can yield a formulation that is retained on the mucosal membrane where the drug is to be delivered. Therefore, a matrix formed by chitosan acts as a balanced drug-delivery system and sustains the drug release [15]. Hence, chitosan proved to be effective in the treatment of various diseases over a prolonged period of time by fixing the drug (with its enhanced mucoadhesive properties) at the site of application. In contrast to our earlier study, a comprehensive assessment was carried out to explore how best to enhance the therapeutic delivery of sitagliptin-loaded chitosan nanoparticles through prolonged retention at the site of absorption, thereby improving its clinical efficacy. Spray-drying was used to synthesize the nanoparticles, which delivered SCN orally, to treat patients with diabetes. Chitosan was used in the formulations to promote the bonding of the drug to the mucosal layer, so that a matrix could form and prolong the drug-release time.

## 2. Materials and Methods

Sitagliptin, chitosan, acetic acid sodium deoxycholate (purity ≥ 97%), and membrane filters 0.22 *µ*m in size were purchased from Sigma-Aldrich Co. (St. Louis, MO, USA). The reagents used were of high-performance liquid chromatography (HPLC) grade. Double-distilled deionized water was used for the experiments.

### 2.1. Formulation of the SCN Nanoparticles

A total of 1,000 mg each of SCN and chitosan were taken and combined in 250 mL of deionized water. The solutions were made to a pH level of 5; acetic acid (0.25% [v/v]) was used to prepare the solution for spray drying. The spray-dried nanoparticles were developed using a spray dryer in a laboratory setting (Buchi B-90, Labortechnik AG, Flawil, Switzerland). The process set at an inlet temperature of 100°C with a rate of aspiration of 100%, a spray flow rate of 160 L/hour, and the pump was adjusted to 2.5 mL/minute. These balanced the outlet temperature of 35°C. A collecting vessel was used to collect the dried powders and the yield rate was recorded. All experiments were performed in triplicate.

### 2.2. Scanning Electron Microscope (SEM)

The spray-dried microspheres were observed under a scanning electron microscope (EVO MA15, Zeiss, Oberkochen, Germany) at a high vacuum of 20 kV. The spray-dried nanoparticle samples were sputter-coated with an ultrathin layer of electrically conducting metal (gold) in a partial vacuum. Micrographs were observed, captured, and imaged.

### 2.3. Nanoparticle Size Measurement

The size of each particle was measured using a Malvern Zetasizer Nano ZS (Malvern Instruments, Malvern, UK). The particles were kept in absolute ethanol for dispersion (refractive index: –2.43) and were measured at room temperature.

### 2.4. Swelling Behavior Study

The swelling behavior of the spray-dried nanoparticles was carried out in simulated gastric fluid (pH 1.2). The dried nanoparticles sizes were measured under a microscope after the nanoparticles were dipped in gastric fluid (pH 1.2) at intervals of 5 minutes, 15 minutes, 30 minutes, 1 hour, 3 hours, 5 hours, 8 hours, and 12 hours. The percent swelling was calculated using the formula

(1) $$\begin{array}{c} Swell\left[\%\right]=Mt-\frac{M0}{M0}\;\times 100,\\ \text{where time}\text{“}t\text{”}=\left[Mt\right];\text{ initial time }\left[t=0\left[M0\right]\right]. \end{array}$$

### 2.5. Production Yield

The nanoparticles were collected from the spray-drying chamber and the total nanoparticle yield was measured. The following formula was used to calculate the yield:

(2) $$\begin{array}{c} \%\text{ yield}=\frac{\left[\text{actual yield}\right]}{\left[\text{theoretical yield}\right]}\times 100. \end{array}$$

### 2.6. Wash‐Off Test (In Vitro)

The Ranga Rao and Buri [23] method was used to determine the adhesiveness of the nanoparticles. Rats were anesthetized using isoflurane/O_2_. The mucosal layer of the stomach (∼5 cm in length, 1 m in width) was neatly cut and washed in normal saline. Then, 100 mg of the drug was carefully placed on the mucosal surface and allowed to interact for up 30 minutes [24]. The mucus layer was placed in a desiccator for up to 15 minutes; the humidity was set at 90%. The mucosal layer was washed in saline at a flow rate of 5 mL/minute. The concentration of the drug was read in a spectrophotometer.

### 2.7. In Vitro Drug Release and Kinetics of the SCN Formulation

The in vitro drug release of the SCN formulation in the solution was studied sequentially at a pH of 1.2 and a pH of 6.8 to stimulate fasting pH levels in the stomach and intestine, respectively. The prepared nanoparticles were briefly suspended in the solution at a concentration of 0.1% (w/v); then, the solution was incubated in an orbital shaker at 37°C at 100 rpm. Following incubation for 30 minutes, 1 hour, and 2 hours at a pH of 1.2 and for 30 minutes, 1 hour, 2 hours, 4 hours, 8 hours, and 12 hours at a pH of 6.8, a 100 *µ*L sample was aliquoted and adjusted with an equal volume of fresh medium accordingly. Each aliquot was then centrifuged (3,000 rpm for 15 minutes) in a plastic tube and the supernatant was separated to further determine the SCN released by ultraviolet (UV) spectrophotometer at 267 nm. The obtained results were compared with different kinetic models to better understand the mechanism of drug release.

### 2.8. FTIR Spectra of Sitaglitpin, Chitosan, and the Formulation

The absorption spectra were obtained in the range of 500–4,000 cm^−1^ using an attenuated total reflectance (ATR) FTIR (8400, Shimadzu, Kyoto, Japan). Samples were put on the sample holder and the distance between the sample holder screw and zinc selenide crystal was adjusted to obtain 64 scans following a background correction.

### 2.9. DSC Analysis of Sitaglitpin, Chitosan, and the Formulation

A DSC analysis was performed using a differential scanning calorimeter (DSC 1, Mettler-Toledo, Columbus, OH, USA). The samples were sealed in standard aluminum pans and heated from 40°C to 300°C at a heating rate of 10 K/minute under nitrogen gas and purged at a 40–60 mL/minute flow rate.

### 2.10. Drug Distribution in the GIT

Male Sprague–Dawley rats weighing 250–300 g, and which were fasted for 20–24 hours before the experiment, were selected. The rats were divided into five groups of 15 rats. The control rats (the first group) were given the SCN solution orally. The second to fifth sets received the SCN nanoparticles. Orally administered nanoparticles were injected nonanesthetically using a rubber tube at a dilution of 25 mg (nanoparticles)/1 mL (saline). Three rats were sacrificed from each group following 1 hour, 3 hours, 5 hours, 8 hours, and 12 hours of administration. The stomach and small intestine were taken together (Section 1) and subdivided into six sections (Sections 2–7; 14 cm in length each). These were cut open, so that the mucosal layer would become exposed. The nanoparticles were collected using a spatula by neatly removing the layer. SCN was extracted by homogenization and the mashed solution was incubated for 24 hours; it was then spun at 1,000 × g for 30 minutes. The supernatant was spectrophotometrically analyzed.

## 3. Results and Discussion

The mucoadhesive SCN nanoparticles had structures that differed in terms of outer-layer texture and drug-loading patterns, which depended on different methods to create various formulations. The drug loading was around 72.5% ± 5%, whereas the nanoparticle content was 84.9% ± 3% in 500 mg of powder, which is relatively higher than the data reported in our earlier study [24]. Product was lost while loading the drug, as the nanoparticles were left behind in the spraying chamber or were lost during manual collection from the electrode [11].

**Figure 1:**
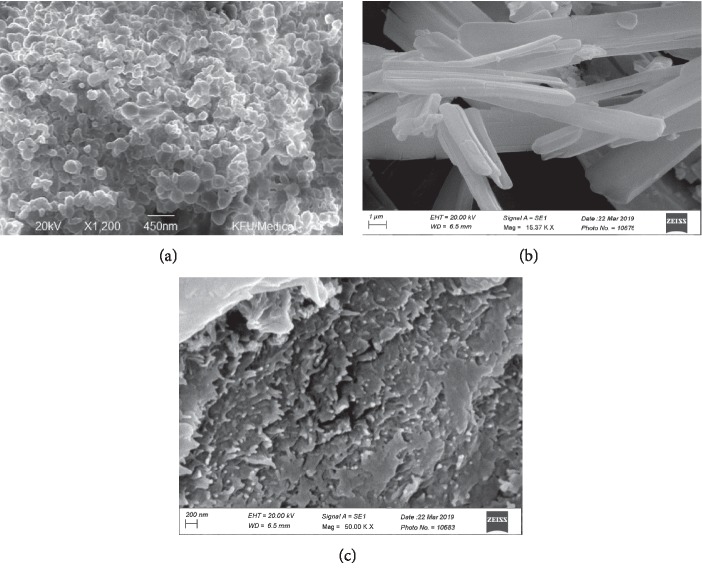
SEM image showing the formulation (a), pure drug (b), and the chitosan (c).

The developed nanoparticles demonstrated an almost spherical scattering pattern, which occurred in a rhythmic manner. There was no evidence of any contractions on the surface (Figure 1(a)), and the nanoparticles were compared with the pure drug (Figure 1(b); sitagliptin) and polymer (Figure 1(c); chitosan). Uniform spray drying was observed for all spheres. The nanoparticles differed in shape due to the varied moisture content and the drying temperature used. Specifically, the smaller the particle, the faster it was able to dry.

**Figure 2:**
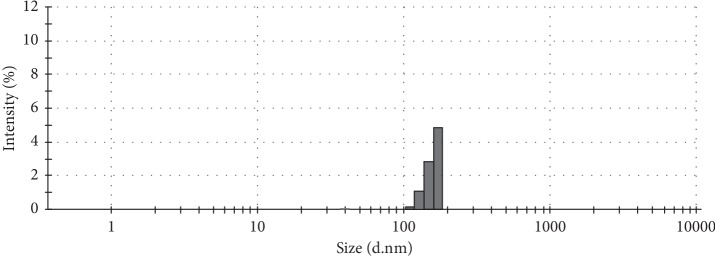
Nanoparticle size plotted against intensity (%). The average size of the particles was ∼350 nm.

Our scanning electron microscopy (SEM) results varied in accordance with the size of the SCN nanoparticles (Figure 2), with a mean size of 350 nm; smaller sizes were found to be 210 nm and larger sizes were 618 nm. However, these data signify that the particle sizes are relatively small when compared with the data observed in our earlier study [24]. Typically, small particles will enhance the total surface area given their narrow size. Narrowing of the nanoparticle size indicated abrupt SCN release at the absorption site; thus, this was associated with the sustained release of a considerable amount of SCN and demonstrated the hypoglycemic effects of this drug.

**Figure 3:**
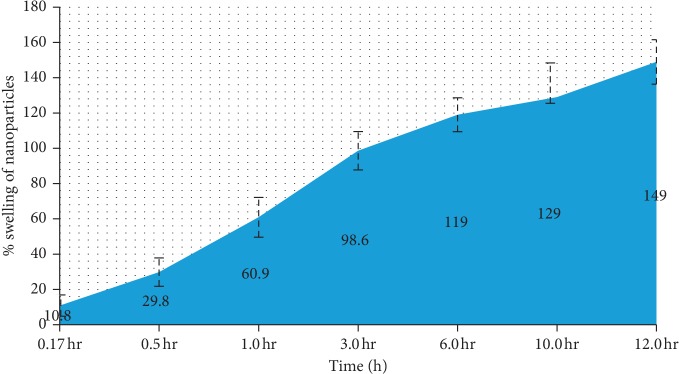
Swelling percentage of the nanoparticle formulation. Particle swelling began within 7 minutes, and a gradual increase was noticed given the prolonged time spent on the mucosal membrane.

The swelling percentage of the SCN nanoparticle formulation was also monitored at different time intervals (Figure 3). It was observed that all nanoparticles rapidly swelled within 7.2 minutes in saline at a pH level of 1.2. The mucoadhesiveness of the nanoparticles was reported earlier, and the cohesiveness of the polymers was represented by the swelling index [25]. Overall, it was found that liquid in the stomach was absorbed by the nanoparticles. The capillary action of the mucosa contributes to stronger adhesion. SCN formulation swelling was around 168% ± 15%.

Bioadhesion studies were carried out to understand the nature of mucoadhesion. Our results were validated by the significant increase in SCN nanoparticles ex vivo (31.5 ± 0.25 hours). This was due to the electrostatic bond between the nanoparticles and glycoprotein in mucin. The primary reaction observed was between the hydrogen bond, the electrostatic attraction of the nanoparticles, and the hydrogen atoms of mucin, thus resulting in strong adhesive forces among the nanoparticles. This mucoadhesion formed the basis for the systematic release of SCN from the nanoparticles into the GIT. SCN bursts directly from the intestinal mucosa in rats in the control group due to the absence of adhesiveness.

**Figure 4:**
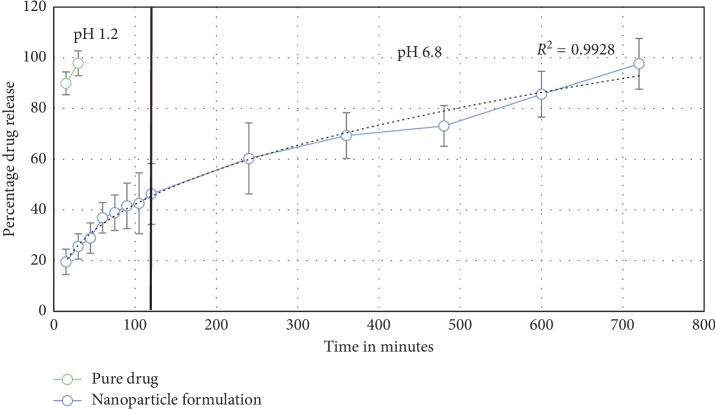
In vivo release comparisons in the stomachs of Sprague–Dawley rats following oral administration of the pure drug and nanoparticle formulation.

Different from our earlier study, the profiles of the SCN drug release formulations in vitro were assessed while maintaining the different solutions at pH levels of 1.2 and 6.8 for 2 hours and 12 hours at 37°C, which are similar to the pH levels of the stomach and small intestine, respectively (Figure 4). There was an initial burst in the first hour due to the desorption of SCN at the top of the chitosan nanoparticles, followed by a delay in drug release. The amount of SCN released was found to be around 45.1% at 2 hours (pH 1.2), showing that the SCN was loaded deep inside the nanoparticles, facilitating the release of SCN into the stomach while fasting. Therefore, an initial burst favors the maintenance of a constant concentration of the therapeutic drug at pH 1.2. At pH 6.8, the release of SCN was 98.1% over the next 12 hours. These results revealed the systematic and perfect release of SCN nanoparticles.

Our experimental results from this in vitro study were compared with different kinetic models, such as those of Baker and Lonsdale (first order); Weibull, Hixson, and Crowell; and Peppas and Higuchi [24, 26]. Sigma Plot (Ver. 9.01) was used different mathematical models to determine the release mechanism. The results best fit with the Peppas model, with a correlation coefficient of *R*^2^ = 0.9928. The fit with the Peppas model indicates that the release of SCN from the nanoparticles occurred by a polymer matrix and obeyed Fick's law of diffusion, which transported the drug from the polymeric matrix. The mucoadhesion of the nanoparticles, which was characterized by the initial swelling and adsorption of (or the weak binding to) the surface of polymeric nanoparticles quite likely contributed to the high rate of “burst” [27]. After this burst cascade, the controlled release of SCN from the nanoparticles was maintained for many hours in the stomach.

**Figure 5:**
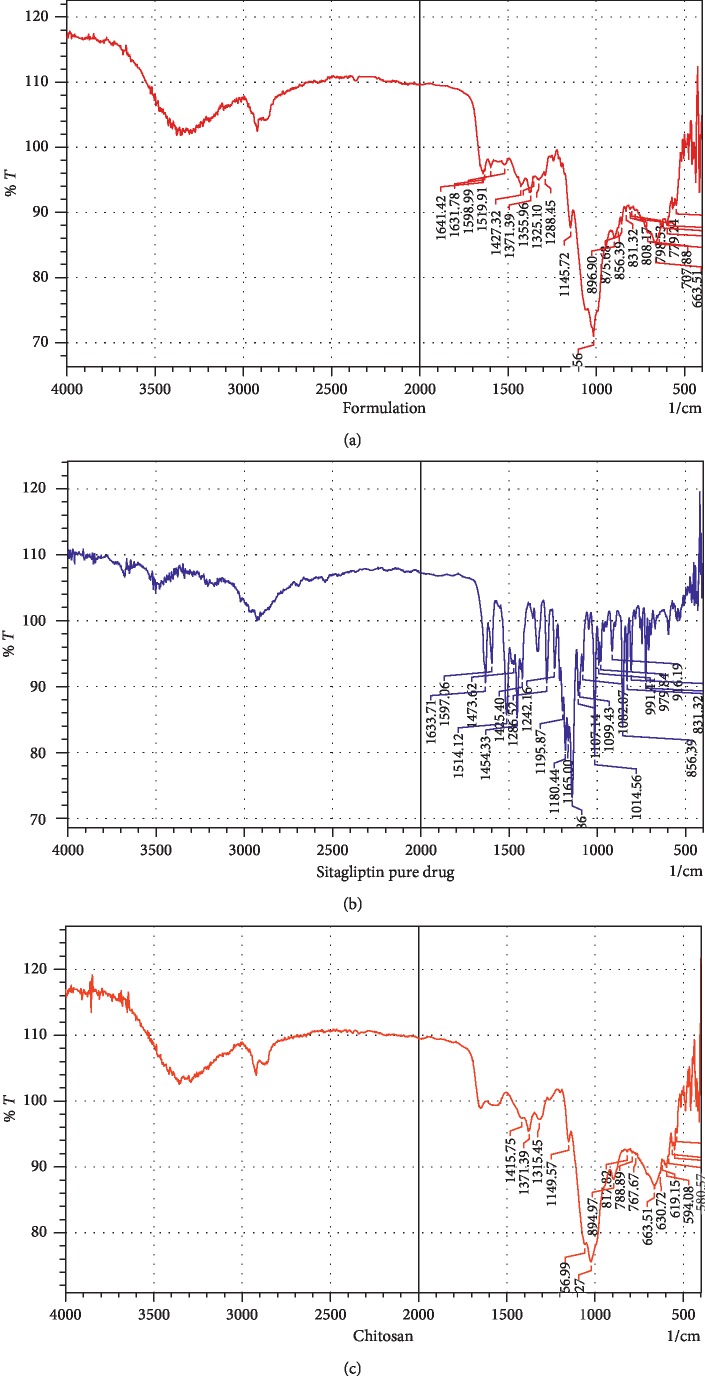
The FTIR spectra of the pure drug formulation (a), sitagliptin (b), and chitosan (c).

The FTIR spectra of the pure drug showed significant bands at 3,339.06 (amine functional group), 3,149.80 (C–H stretching), and 1,633.71 cm^−1^ (C=O group); N–H bending occurs around 1,597.06 cm^−1^ for primary amides, while for secondary amides it occurs around 1,514.12 cm^−1^. In terms of the major characteristic peaks, those for pure chitosan appeared around 1,141.75 cm^−1^, 1,371.79 cm^−1^, and 1,315.41 cm^−1^, corresponding to the presence of O–H bending, C–O stretching, C–N stretching, C–O–H bending (1,415.75 cm^−1^). Further, a significant band was observed at 3,794.23 cm^−1^ for aromatic C–H stretching. Similar significant characteristic peaks of the drug remain unchanged in the formulation (Figure 5(a)), which confirms that there was no significant interaction between SCN (Figure 5(b)) and the polymer (Figure 5(c)).

**Figure 6:**
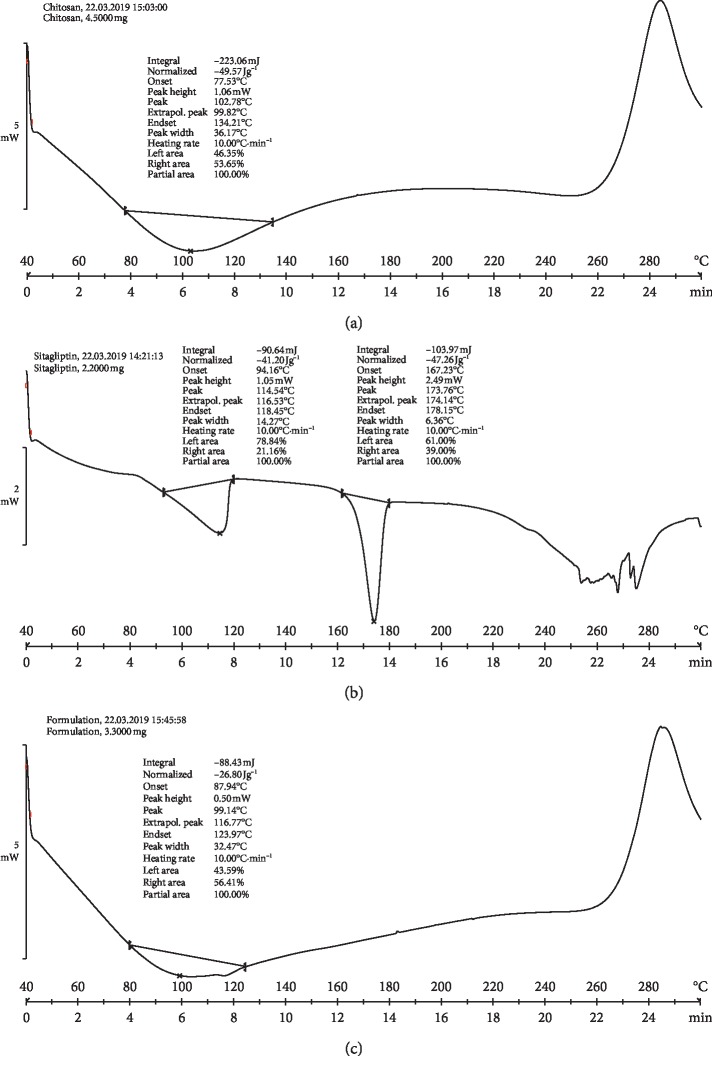
DSC analysis of the pure drug (sitagliptin (a), chitosan (b), and the formulation (c)).

The DSC thermograms of the endothermic peak onset temperatures for pure chitosan appeared at 102.78°C, while the broadened peak endset temperature occurred at 134.21°C (Figure 6(a)). However, the physical mixture of chitosan and the drug exhibited a peak onset temperature at 94.16°C and a peak endset temperature of 118.45°C; the actual melting point of the drug had an onset peak temperature of 167.23°C and a peak endset temperature at 178.15°C, respectively (Figure 6(b)). Further, an examination of the DSC analysis of the formulation showed that the drug peak disappeared given that the drug may have been in an amorphous state or a disordered crystalline state (Figure 6(c)).

Sprague–Dawley rats were orally administered SCN suspensions and SCN nanoparticles. The drug concentration percentage was read as previously described. At regular intervals of 0.5 hours, 1 hour, 4 hours, 6 hours, 8 hours, and 12 hours, the mucoadhesiveness of the particles was studied in vivo. The results revealed that the percent of SCN nanoparticles retained in the stomach was significantly higher than the percent of SCN suspension retained (*P* < 0.05), likely due to the narrow particle size of the prepared nanoparticles.

**Figure 7:**
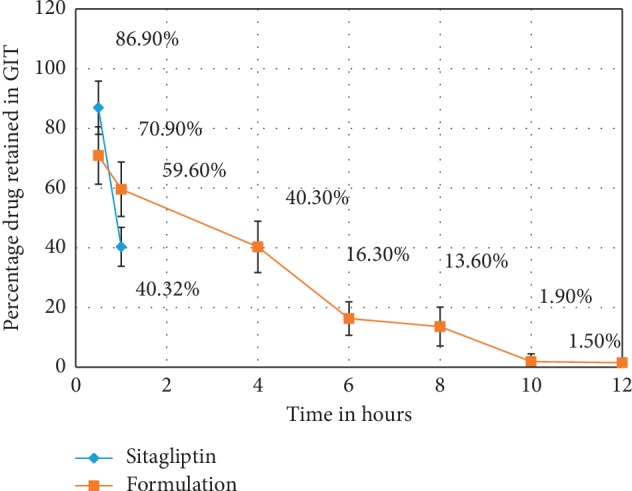
In vivo release comparisons in the stomachs of Sprague–Dawley rats following oral administration of the pure drug and nanoparticle formulation.

The polymer was deep inside the nanoparticles and had swollen into a large mass of polymers at a pH level of 1.2 (swelling behavior). As such, a large surface area had formed, and the interaction between the nanoparticle and mucin was conducive to prolonged residence in the gastrointestinal tract. Another reaction that favors adhesion is hydrogen bonding [11, 28]. The chitosan polymers primarily adhered due to the hydrogen bond, the hydrophobic interactions with gastric mucin, the presence of amino groups, and electrostatic forces. The release percentage of SCN after 30 minutes was 29.1%, while at 1 hour it was 40.1%, at 4 hours it was 59.7%, at 6 hours it was 83.7%, at 8 hours it was 86.4%, at 10 hours it was 98.1%, and at 12 hours it was 98.4% (Figure 7). The percentage of the pure drug released after 30 minutes was around 40.32%, and after 1 hour it was 86.9%. Additionally, the SCN nanoparticles exhibited 29.1% drug release in 30 minutes, which means that a significant amount of SCN was weakly bound to or adsorbed on the surface of the nanoparticles. This is likely due to an “initial rapid release” and supports the notion that the formulation exhibits rapid and significant therapeutic effects on patients in clinical settings. When compared with the pure drug, the SCN nanoparticles showed a sustained release pattern.

## 4. Conclusions

Based on our results, we were able to conclude that a nanoparticle with mucoadhesive properties could be optimized, and this formulation was developed to ensure that SCN could remain in the stomach over a prolonged period of time. The nanoparticle-based mucoadhesive substances investigated here represent a novel form of drug administration that is ideal for patients with T2DM.

## Data Availability

The data from the SEM, particle size, in vitro release, FTIR, DSC, swelling, and in vivo release studies that were used to support the findings of this study are included within the manuscript.
